# Strong Selection Against Early Generation Hybrids in Joshua Tree Hybrid Zone Not Explained by Pollinators Alone

**DOI:** 10.3389/fpls.2020.00640

**Published:** 2020-05-26

**Authors:** Anne M. Royer, Jackson Waite-Himmelwright, Christopher Irwin Smith

**Affiliations:** ^1^Biology Department, The University of Scranton, Scranton, PA, United States; ^2^Biology Department, Willamette University, Salem, OR, United States

**Keywords:** *Yucca*, Joshua tree, *Tegeticula*, geographic clines, hybrid zone, coevolution

## Abstract

Coevolution frequently plays an important role in diversification, but the role of obligate pollination mutualisms in the maintenance of hybrid zones has rarely been investigated. Like most members of the genus *Yucca*, the two species of Joshua tree (*Yucca brevifolia* and *Yucca jaegeriana)* are involved in a tightly coevolved mutualism with yucca moths. There is strong evidence of a history of coevolution between Joshua trees and their moth pollinators. We use a geographic clines approach in the Joshua tree hybrid zone to ask if selection by the moths may currently contribute to maintaining separation between these species. We compare genomic, phenotypic, and pollinator frequency clines to test whether pollinators maintain the hybrid zone or follow it as passive participants. The results reveal dramatic overlapping genomic and pollinator clines, consistent with a narrow hybrid zone maintained by strong selection. Wider phenotypic clines and a chloroplast genomic cline displaced opposite the expected direction suggest that pollinators are not the main source of selection maintaining the hybrid zone. Rather, it seems that high levels of reproductive isolation, likely acting through multiple barriers and involving many parts of the genome, keep the hybrid zone narrow.

## Introduction

Understanding the forces that drive speciation has been a question of interest since the field of evolutionary biology began, and continues to generate complex questions, including the role of intrinsic and ecological factors (Coyne and Orr, 2004; Sobel et al., 2010; Nosil, 2012). The importance of intrinsic isolation, including Bateson–Dobzhansky–Müller incompatibilities in the later stages of speciation, is well established (Coyne and Orr, 1989, 1997) and the role of ecology has attracted particular interest in recent years (Schluter, 2000; Nosil, 2012). In seeking to understand the final stages of speciation, hybrid zones provide a particularly valuable resource—laboratories of speciation where the full range of forces (including genetics, geography, and ecology) can act and interact to shape the trajectory of diversification (Hewitt, 1988; Barton and Hewitt, 1989). Pollinator mediated selection, particularly within obligate pollination mutualisms, has been suggested as an important mechanism by which ecological factors may promote speciation and reproductive isolation (Kiester et al., 1984; Armbruster and Muchhala, 2008), but hybrid zone analyses have rarely been used to test this idea. Examining hybrid zones and clinal variation within presents a rich framework in which to understand both speciation and the potential role of pollinators in promoting plant diversification.

Ecological forces contributing to speciation can be divided into biotic and abiotic forces, with both playing important and varying roles depending on the system (Coyne and Orr, 2004; Lowry et al., 2008; Nosil, 2012; Baack et al., 2015). Interactions between species are particularly interesting because of the potential for the species to both evolve in response to each other (Janzen, 1980), offering opportunities for direct and diffuse coevolution. Such coevolution can contribute to explaining phenomena involving speciation including adaptive radiations (Ehrlich and Raven, 1964; Kiester et al., 1984; Smith and Benkman, 2007; Althoff et al., 2014; Hembry et al., 2014; Marquis et al., 2016) and the latitudinal biodiversity gradient (Mittelbach et al., 2007). Although there is abundant evidence of pairwise interspecific interactions spurring speciation, largely in the context of antagonisms (Mitter et al., 1988, 1991; Farrell, 1998; Yoder and Nuismer, 2010), the potential for mutualisms to foster diversification is a matter of debate and interest (Yoder and Nuismer, 2010; Hembry et al., 2014). In spite of a large body of literature on interspecific interactions and speciation, there are relatively few studies examining obligate pollination mutualisms in hybrid zones (but see Leebens-Mack et al., 1998; Moe and Weiblen, 2012).

The genus *Yucca* in general is a useful system for studying speciation and mutualism due to the tight relationship between many *Yucca* species and their moth pollinators (*Tegeticula* spp.). The vast majority of *Yucca* are pollinated exclusively by the obligate mutualist moths, often in a reciprocally obligate relationship (i.e., one species of *Tegeticula* for one species of *Yucca*). *Tegeticula* are nursery pollinators, laying their eggs in the developing fruits of the flowers they pollinate. The resulting larvae exact a cost on the plants, eating many of the developing seeds. In many of these plant-moth pairs, there is phylogenetic evidence of co-speciation, with the patterns of divergence in moths and plants frequently matching (Althoff et al., 2012). This raises the question of whether the mutualism contributes to speciation or simply causes one mutualist to follow its partner in diversification (Althoff et al., 2014). One group that shows promise for addressing these questions is Joshua trees and their pollinators.

**FIGURE 1:**
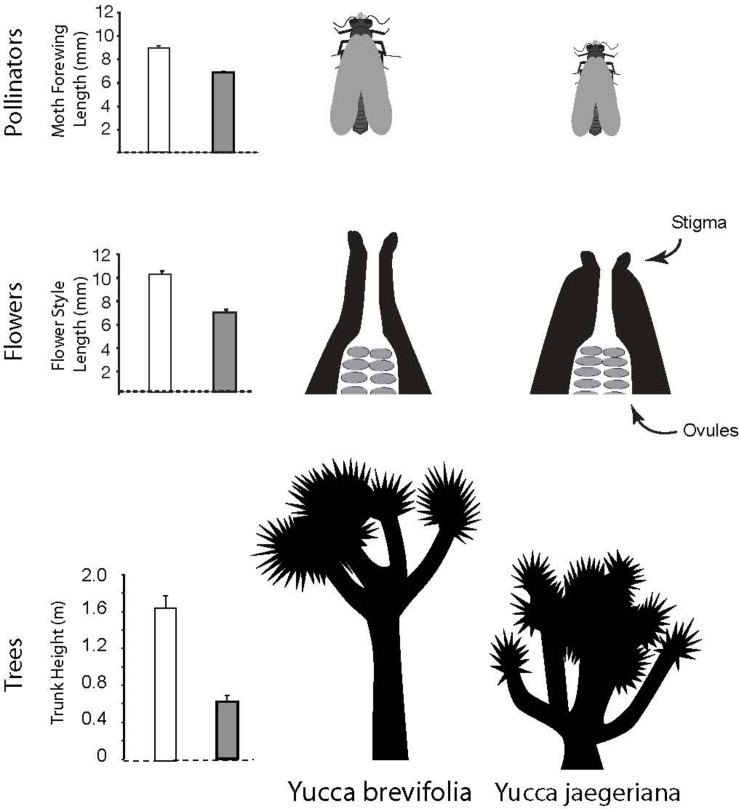
Variation in body size in Joshua tree moth pollinators (graph illustrates a common proxy, wing length), and variation in two key characters differentiating the two species of Joshua tree: style length and trunk height.

Joshua trees (*Yucca brevifolia* and *Yucca jaegeriana*) offer the opportunity to fill this gap, taking advantage of natural variation in both mutualists across an existing hybrid zone (Rowlands, 1978; Smith et al., 2008) to understand how the interspecific interaction contributes to late-stage speciation. In Joshua trees, there is additional evidence that the sister species (*Y. brevifolia* and *Y. jaegeriana*) and their moth pollinators (*Tegeticula synthetica* and *Tegeticula antithethica*) have coevolved (Pellmyr and Segraves, 2003; Godsoe et al., 2008; Smith et al., 2008). Work on coevolution in these taxa has focused on two key characters that appear to be integral to the mutualism: style length in the trees and body size in the moths (Godsoe et al., 2008; Cole et al., 2017) (Figure 1). Of a suite of phenotypic traits measured in the trees, style length (the distance between the stigma and the ovules, and the site of moth oviposition; Trelease, 1893; Cole et al., 2017) is one of the most consistently and dramatically differentiated between *Y. brevifolia* and *Y. jaegeriana*, with trunk height being just behind (Godsoe et al., 2008) or slightly more strongly differentiated (Royer et al., 2016). Most importantly, style length and moth ovipositor length are strongly correlated in comparisons across species (with *Y. brevifolia* having long styles and *T. synthetica* a larger body) (Godsoe et al., 2008, 2010; Yoder et al., 2013). This pattern of trait matching, consistent with coevolution, may also be present (although weaker) in intraspecific variation (compare Godsoe et al., 2010 vs Yoder et al., 2013).

The existence of a hybrid zone between *Y. brevifolia* and *Y. jaegeriana*, located in Tikaboo Valley in southern Nevada (Starr et al., 2013; Royer et al., 2016), has made more detailed studies of potential coevolution and the dynamics of speciation between this species pair possible. Data on pollinator fidelity and performance on the alternate host show that both moth species prefer their normal host and have lower fitness on the alternate, which may be related to phenotype matching (Smith et al., 2009). There is also asymmetry in crossing success, with *T. antithethica* found more frequently, and producing more offspring, on the “wrong” host than *T. synthetica* (Smith et al., 2009). This is expected to result in gene flow from *Y. jaegeriana* into *Y. brevifolia* via pollen. Such gene flow is supported by population genetics work using microsatellite markers in and around the hybrid zone indicating nuclear gene flow into *Y. brevifolia* (Starr et al., 2013), as well as range wide chloroplast data (Smith et al., 2008) showing *Y. brevifolia* chloroplasts moving into the *Y. jaegeriana* nuclear background (presumably as they are swamped by *Y. jaegeriana* pollen). Additionally, analyses of single nucleotide polymorphisms associated with trait variation show strong divergent selection in allopatry and disruptive selection in the hybrid zone on style length, as expected if style length is a key trait in divergence (Royer et al., 2016). Thus, key pieces of ecological and population genetic data are consistent with the pollination mutualism playing a role in diversification.

However, other pieces of evidence cloud the picture, suggesting that the pollinators are close followers rather than drivers of speciation in the Joshua tree hybrid zone. The two *Yucca* species are highly genetically differentiated, with F_ST_ across the hybrid zone estimated at 0.29–0.30 (Royer et al., 2016). This level of differentiation in close proximity supports strong reproductive isolation, in spite of range-wide microsatellite and chloroplast data suggesting a long history of gene flow between species (Smith et al., 2008; Yoder et al., 2013). Range wide nuclear data show gene flow primarily from the hybrid zone into *Y. jaegeriana*, opposite the direction predicted by pollinator behavior (Yoder et al., 2013) and gene flow found in proximity of the hybrid zone (Starr et al., 2013). Finally, the same analysis that showed strong disruptive selection on style length also showed the same results for other strongly differentiated traits in and around the hybrid zone, particularly trunk height (Royer et al., 2016). In order to disentangle how the suite of forces acting in the Joshua tree hybrid zone are shaping the final stages of speciation, a new approach is clearly required.

**FIGURE 2:**
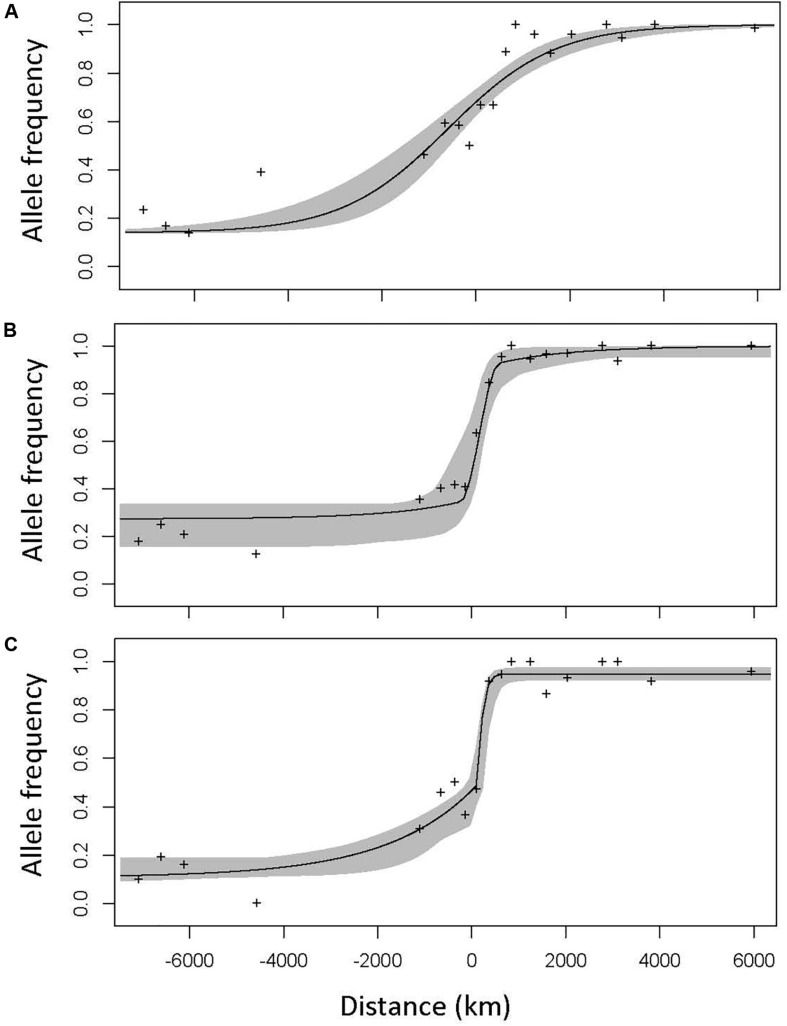
The three cline model types, illustrated using the clines of high—F_ST_ SNPs in the Joshua tree hybrid zone. The center of the hybrid zone (0.5 hybrid contour, determined using microsatellite genotypes) is marked zero. Model I **(A)** is characterized by a gradual, symmetrical cline. Model II **(B)** is a narrower cline with long, symmetrical introgression tails, characteristic of strong selection against hybrids in the early generations and/or in the center of the hybrid zone, but with portions of the genome escaping and successfully introgressing into both parental backgrounds. Model III **(C)** shows similarly strong selection, but with substantially more introgression in one direction (in the case of the Joshua tree hybrid zone, that direction is always to the west—from *Y. jaegeriana* into *Y. brevifolia*). A null model would be a perfectly straight line from the allele frequency on the western edge to the allele frequency on the eastern edge, often (but not necessarily) with slope zero.

Geographic clines analyses, which quantify how allele frequencies and phenotypes shift as one moves across a hybrid zone, can help discern what evolutionary and ecological processes are currently most important in shaping the hybrid zone (Barton and Hewitt, 1985; Mallet et al., 1990; Stankowski et al., 2017). This explicitly spatial approach can give us insight into how selection changes across the hybrid zone: which traits and parts of the genome are under stronger selection, how the distribution of the moth species changes across the hybrid zone, and how moths and trees interact. If taxa that diverge in allopatry come back into contact and hybridize, there are several possible evolutionary outcomes. If there is no reproductive isolation, no selection against hybrids, one expects abundant gene flow between the parental taxa with unimpeded symmetrical introgression. This creates a non-clinal pattern; a figure depicting change in phenotype or allele frequency over space would be a straight line with constant slope, and over many generations the hybrid zone would broaden and eventually disappear (Barton and Hewitt, 1985). On the other hand, if hybrids are less fit, selection will favor a quick transition over space from one parental taxon to another, producing a cline—a line depicting allele frequency or phenotype changes over space that will be flat at the edges of the hybrid zone, and then suddenly assume a steep slope at the center (Figure 2). The stronger the selection, the narrower the cline (the transition happens over a smaller space). The center of the cline—the inflection point where the slope is the steepest—then indicates the geographic location where selection switches from favoring the genotype/phenotype of one parent to the other. Stepped clines, with particularly steep centers and long introgression tails, indicate particularly strong selection against early-generation hybrids and/or hybrids in the geographic center of the hybrid zone, with only sections of the genome separated by recombination successfully introgressing. Consistent selection against hybrids will thus result in a narrow straight tension zone, with many coincident clines, which is expected if speciation is advanced and selection is limiting introgression across the genome (Key, 1968; Barton, 1979; Barton and Hewitt, 1985; Buggs, 2007).

Here, we use a geographic clines approach to analyze changes in moth abundance, nuclear and chloroplast genomic markers, and phenotypic traits across the hybrid zone to understand the process of speciation in Joshua trees. First, we hypothesize that selection for differentiation in style length, driven by coevolution with moth pollinators, is maintaining the separation of the species. We predict that strong selection from the pollinators will result in the geographic cline for style length being narrower than the clines for other traits, and closely coincident with the distribution of the moths. We expect geographic clines for SNPs associated with variation in style length to show the same pattern, coinciding closely with the distribution of moths shifting across the hybrid zone when compared to the genome-wide average and SNPs associated with other traits. Second, we hypothesize that trait mismatch between moths and floral features results in asymmetry in the direction of successful crosses, which should impact cytoplasmically inherited genomic material differently from the nuclear genome. Accordingly, we predict chloroplast haplotype clines should be shifted east relative to nuclear SNP clines, consistent with existing data on pollen flow and pollinator behavior.

## Materials and Methods

**FIGURE 3:**
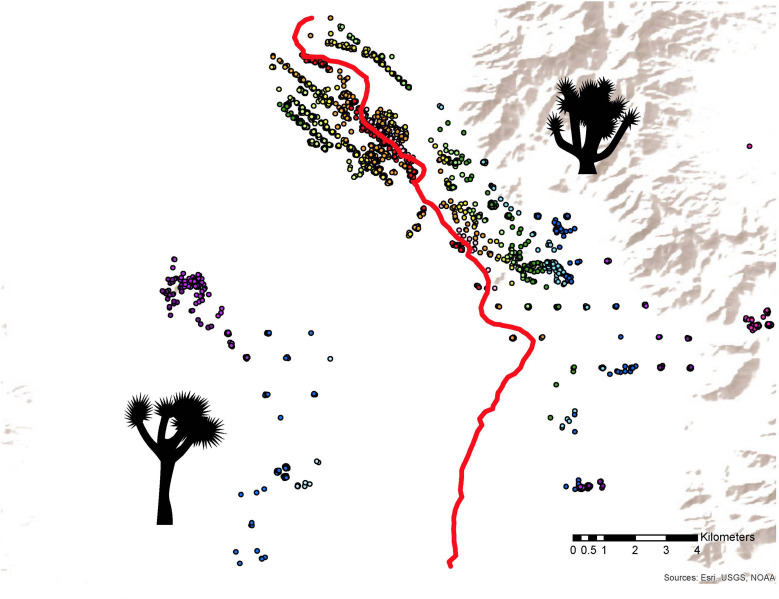
Sampling distribution in the hybrid zone. *Y. jaegeriana*, typified by short trunks and short styles, are located in the east; *Y. brevifolia*, with tall trunks and long styles, are located in the west. Trees are color coded by distance from the center of the hybrid zone (red line), e.g., all green points represent trees that are a similar distance from the 0.5 hybrid contour. There are vegetative and microsatellite data for all trees depicted here; a subset also have floral and/or SNP data (see Supplementary Figure S1).

Genotype and/or trait data were collected from a total of 1734 trees in the hybrid zone and nearby allopatric areas in Tikaboo Valley, Nevada, United States (37.46°N, 115.5°W), with heaviest sampling in the hybrid zone (Table 1 and Figure 3). Trees were sampled across the entire known hybrid zone, with sampling most intense near the center; a gap in sampling west of the hybrid zone represents the discontinuous distribution of trees rather than undersampling (Figure 3). Variation in floral and vegetative traits between species has been described previously (Godsoe et al., 2008; Royer et al., 2016). We used geographic cline analysis to look at the spatial distribution of hybrid zone variation in traits that differ significantly between the two species, including branch number, tree height, leaf length and width, trunk height, petal length and width, pistil length and width, style base width, and style length. Because Joshua trees do not flower each year, fewer trees have measurements for floral traits than trunk height (Table 1 and Supplementary Figure S1). For details on how traits were measured, see Royer et al. (2016).

**TABLE 1:** Number of trees sampled from locations outside the hybrid zone (indicated with species name) and within the hybrid zone.

Cline	*Y. brevifolia*	Hybrid zone	*Y. jaegeriana*	Total
**Vegetative traits**				
Branch number	288	2353	240	2881
Leaf length	278	2398	241	2917
Leaf width	288	2398	243	2929
Trunk height	254	1783	231	2268
Height	288	2398	235	2921
**Floral traits**				
Petal length	36	424	61	521
Petal width	36	424	61	521
Pistil length	36	426	63	525
Pistil width	36	426	63	525
Style length	36	426	63	525
Style base width	36	426	63	525
**Genomic**				
Chloroplast genotypes	59	127	29	215
SNP genotypes	62	183	63	308
**Pollinator**				
Moth sp. frequency	16	72	13	101

*The SNP category covers SNP Qscores as well as high F_*ST*_, random SNPs, and analyses of loci associated with trait variation.*

To estimate the distribution of moths, sticky traps were placed on flowering trees (including members of both parental species and hybrids) across the zone of sympatry during the springs of 2011–2013 and 2015. In each year, the sticky traps were left in place for the length of the flowering season. *Tegeticula* moths stuck to the traps were identified to species using diagnostic variations in the cytochrome oxidase gene sequenced in one direction from the 5′ end using Sanger sequencing by Eurofins (Louisville, KY, United States) (Pellmyr and Segraves, 2003; Smith et al., 2008, 2009).

Leaf tissue for genotyping trees was collected in the field and dried with silica gel, or flash frozen in liquid nitrogen and later transferred to −80°C. DNA was extracted using the Qiagen (Germantown, MD, United States) DNEasy Plant mini kit. Ten microsatellite loci were amplified from 1469 trees following the protocol of Flatz et al. (2011). RAD-seq data were produced as described in Royer et al. (2016), resulting in 9516 SNPs at 4000 loci. Because of the greater time and expense involved in generating RADseq data, these SNPs were genotyped in a smaller, but still substantial number of trees (319) sampled in and immediately around the hybrid zone. Previous work in *Yucca* using sequence from five chloroplast genes found no variation in the hybrid zone (Smith et al., 2008). To identify variation in the chloroplast genome within the hybrid zone, we compared whole chloroplast genome resequencing data from populations near the hybrid zone (Smith et al., in review). We identified a number of polymorphic regions that showed promise of carrying species-specific variants based on 16 trees previously genotyped. These included two short regions that were readily amplified by PCR and that contained one SNP and two variable number tandem repeats (VNTRs). We then genotyped 215 trees at those loci. Primers were identified using the consensus chloroplast genome sequence (first set of primers: cpF4416, 5′-CCA-TTG-TCA-ATA-TGA-GTG-GG-3′; cpR4581, 5′-AAG-GAC-GAA-CCT-TGC-TTA-TT-3′. Second set of primers: cpF9932, 5′-TAT-ACG-TTC-TCG-CGA-TTT-GT-3′; cpR10134, 5′-ATT-TGG-CTT-CAA-TCT-TCC-CT-3′). The 200 bp regions were amplified using Qiagen multiplex PCR kits (Qiagen; Hilden, Germany) with 35 cycles of PCR with a 55°C annealing temperature (94°C to denature, 72°C for elongation). The PCR product was purified with ExoSAP-IT (Affymetrix, Inc.; Cleveland, OH, United States) and sequenced at Eurofins MWG Operon USA (Louisville, KY, United States). The sequence was aligned using CodonCode Aligner (CodonCode Corporation^1^), including segments from the consensus chloroplast genome of each species in the alignment. We combined the genotypes at both variant sites to identify chloroplast haplotypes.

### Assigning Individual Trees to Species

Trees were assigned a hybrid index score (identifying parentals and, for hybrids, estimating the proportions of genomes of each tree that came from either species) using the Bayesian clustering software STRUCTURE 2.3.4 (Pritchard et al., 2000). We first confirmed that grouping trees into two species best describes the genetic variation in this region by comparing STRUCTURE runs at levels of K from 1 to 7 (one more than the number of populations sampled). We performed 10 runs per level of K using random seeds with 30,000 burnin followed by 150,000 iterations of MCMC, using the ancestry model with alpha inferred and default parameters (including the admixture model, which produces estimates of proportion genetic identity). Summary statistics for the runs were obtained using STRUCTURE HARVESTER (Earl and vonHoldt, 2012), and indicated convergence across runs at each level of K. We used the ΔK statistic (Evanno et al., 2005) to confirm that K = 2 (corresponding to the two species) is the best fit for our data. Mean probability of a tree belonging to each species was then calculated across the 10 runs at K = 2 using CLUMPAK (Kopelman et al., 2015). We used the proportion of each individual’s genome estimated by STRUCTURE as originating in the eastern species *Y. jaegeriana* as the hybrid index score (*Q*). Qscore*s* calculated using microsatellite genotypes at 10 loci previously shown to be informative in these species (Flatz et al., 2011; Starr et al., 2013) were used to construct the geographic cline transect (see below). This maximized the geographic coverage to get the best estimate of where the center of the hybrid zone is. To calculate the genomic cline itself, we used Qscore calculated using SNPs instead. This reduced the number of trees, but increased our confidence in the estimate of genomic composition for each individual tree.

### Modeling Pollinator Preference

To test the roles of location in the hybrid zone and genetic identity of the tree in shaping pollinator host choice, we performed a Pearson product-moment correlation test to quantify the correlation between Qscore and location in the hybrid zone, and attempted to disentangle the effects of the two variables on pollinator host choice using a general linear model designating the binomial family, with a model: (Frequency of *T. synthetica/T. antithethica*) ∼ Qscore + distance from center of geographic cline transect.

### Constructing the Geographic Cline Transect

To construct a two-dimensional transect for plotting geographic clines, we plotted the GPS coordinates of all sampled trees in ARCGIS ARCMAP 10.3 (ESRI). We used the hybrid index scores to estimate a 0.5 hybrid contour (the center of the hybrid zone) using Empirical Bayesian Kriging (an interpolation method) with prediction and eight sectors. We then estimated the shortest distance between each tree and the 0.5 hybrid contour line using the Near Table tool. The geographic cline of the hybrid zone results from plotting the location of each tree along a single line representing the distance of each from the center of the hybrid zone (the 0.5 hybrid contour line), and then grouping trees that are a similar distance from the center (Figure 3). To produce the final dataset for the geographic cline analysis, trees along the cline were divided into bins set every 250 m along the cline. For all clines (vegetative traits, floral traits, pollinator identity, Qscore, SNPs, and chloroplast haplotype), bins with fewer than three trees for at least one category were collapsed with the closest adjacent bin, producing 19 standard bins used for all cline analyses (Supplementary Table S1).

### Fitting Geographic Clines

#### Genomic Clines: SNPs (Random, High-F_ST_, and GWAS) and Chloroplast Haplotypes

For calculating nuclear genomic clines, we used SNP data previously collected by Royer et al. (2016), described above. When there were loci with multiple SNPs, we reduced the dataset to one SNP per locus because they are expected to be closely linked and result in identical clines. We retained the most statistically significant SNP (with the lowest *p*-value in the F_ST_ or GWAS analysis) when there was a difference, and eliminated one haphazardly when they were statistically indistinguishable. We fitted clines to the SNPs with the highest F_ST_ (top 1%, for a total of 84), using the F_ST_ analysis performed by Royer et al. (2016). We also fitted clines to SNPs significantly associated with the variation in style length (19 SNPs) and trunk height (29 SNPs) in a genome-wide association (GWAS) analysis of the hybrid zone (Royer et al., 2016). We note that there is some overlap in the SNP datasets: four style SNPs and seven trunk height SNPs exhibited high F_ST_, and one SNP was significantly associated with variation in both style length and trunk height. The 100 random SNPs did not include any from the GWAS or high F_ST_ sets. For each cline, allele frequencies for each bin were output directly from the genomic analysis software STACKS (Catchen et al., 2013) in HZAR (Derryberry et al., 2014) format.

For the chloroplast data, we identified only two haplotypes within the hybrid zone. We calculated clinal variation in the frequency of these two haplotypes as described for SNP data above.

#### Trait Clines on Continuous Variables (Phenotypes, Qscores, Moth sp. Frequency)

For phenotypic traits, SNP-based Qscore, and moth abundance data, we standardized the data to a scale from 0 to 1, then ran a linear regression of bin mean trait value on distance along the transect of the hybrid zone. Traits with no significant linear regression were considered to not have clines (i.e., best fit a null model). For those that did change significantly across the hybrid zone, we fitted clines to the data using HZAR, a software package implemented in R that uses the Metropolis–Hastings Markov Chain Monte Carlo algorithm to estimate a curve describing how allele frequencies of phenotypes change across the hybrid zone (Derryberry et al., 2014). Trait distributions were visually assessed for normality (an assumption of HZAR), and transformed when possible to attain normality (tree height was log-transformed, trunk height was square-root transformed). Only moth frequency (a strongly bimodal distribution) was unable to be successfully transformed; we fitted the clines on the untransformed distribution. Distance along the cline for each bin was calculated as the mean distance of all the trees included in the bin.

For all clines, we fitted three models: (I) a simple two-parameter model, setting only a center and width for the cline; (II) a four-parameter model, describing the shape of symmetric introgression tails (β_*0*_/2 and theta0); and (III) six-parameter models with two separate parameters for each asymmetric introgression tail (β_*0*_/2 and theta0, and β_*1*_/2 and theta1) (Figure 2). All models also include a *y*-axis minimum and maximum (pmin and pmax), corresponding to a mean trait value or allele/haplotype frequency in parental populations. For each model type, the parameters and log likelihood of the best-fitting model were used for analysis. For SNP clines, all three models were compared to a null model run in HZAR, which would be a line with constant slope between pmin and pmax (no cline—analogous to the non-significant linear regression described for phenotypic clines above, as HZAR provides no null model for phenotypic clines). We determined the best-fitting model for each trait using AIC values output by HZAR; we selected the model with the lowest AIC, or the simplest model if dAIC < 2.

#### Analysis Comparing Clines

We compared clines by assessing overlap in centers and widths, using the 2 log likelihood (km) estimate (95% credible interval). SNP clines that best fit the null model were excluded from calculations of the mean SNP cline parameters for each category (high F_ST_, random, trunk height, and style length). We then assessed whether there was overlap with the 95% CI of other clines (lack of overlap indicates a significant difference). To evaluate possible sources of selection, we compared cline centers.

### Inferring Levels and Patterns of Hybridization Inside the Zone

Understanding what classes of hybrids are present in a hybrid zone (e.g., F1s, F2s, advanced hybrids, backcrosses) can be helpful in interpreting clines and understanding how advanced speciation is. While hybrid index scores give information about the proportion of the genome originating in each parent, they cannot distinguish between F1s and advanced generation hybrids that maintain equal proportions of the two genetic backgrounds. To do this, it is necessary to incorporate the degree of heterozygosity at each locus into the analysis. When populations are fixed for different alleles at a set of unlinked, biallelic loci, patterns of hybridization can be inferred from hybrid index scores combined with heterozygosities. For example, crossing pure individuals will produce an F_1_ individual that has a hybrid index of 0.5 (50% of alleles inherited from populations and *i* and *j*) will be heterozygous at all loci (*H* = 1.0). Similar analytical predictions can be made for other early generation classes, including backcrossed individuals and hybrid intercrosses (i.e., F_2_s). However, if loci are not completely diagnostic, allele sharing at loci muddies these predictions, making it more difficult to interpret empirical patterns.

Given that the 101 most highly differentiated loci are only semi-diagnostic in these taxa, we used individual-based simulations to generate distributions of multilocus genotypes that are expected to result from hybridization. To do this, we created two groups of “pure” individuals of *Y. jaegeriana* and *Y. brevifolia* based on their *STRUCTURE* (*Q*) scores and geographic distributions. We then used the program hybrid lab (Nielsen et al., 2006) to produce hybrid genotypes by “crossing” individuals from these groups and subsequent hybrid classes *in silico*. We produced an F_1_, an F_2_, an F_1_ backcross to *jaegeriana*, and an F_1_ backcross to *brevifolia.* 500 individuals were simulated for each cross type. For each simulated and empirical individual, we plotted the proportion of heterozygous loci (*H*) on its hybrid index score (HI) defined here as the proportion of alleles coming from pure population *i*. Note that the simulations assume that markers recombine freely (*r* = 0.5), so if some of these loci are tightly linked, we would be underestimating the number of generations of hybridization required to generate hybrid classes beyond the F_1_.

## Results

### Pollinator Occurrence

We found both pollinator species co-occurring in the center of the hybrid zone, over a 4.2 km wide band ranging from ∼1.1 km west of the center to ∼3.1 km east of the center, with 90% of trees with both species observed on them located between 0.4 km west and 2.4 km east. There are instances of moths choosing the “wrong” host, including trees with Q-scores beyond the threshold for identification as “pure” parentals. However, the strong geographic structure in the location of different tree identities leads to a strong correlation between tree location in the hybrid zone and tree Qscore (Pearson’s product-moment correlation = 0.76, *t* = 7.23, *p* = 1.22^∗^10^–8^, *df* = 38). The general linear model showed significant effects of both tree Qscore and location in predicting moth visitation, with Qscore being much more strongly significant (Qscore: *z* = 4.384, *p* = 1.16^∗^10^–5^; location, *z* = 2.16, *p* = 0.03).

### Model Fit and Cline Centers

**FIGURE 4:**
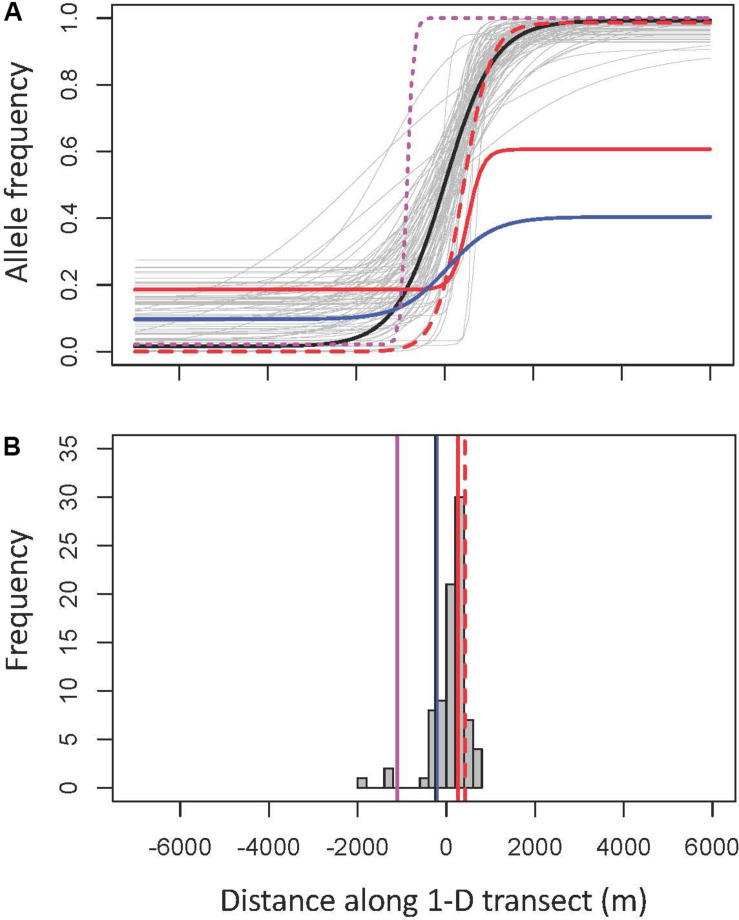
Geographic clines of traits and genotypes in the Joshua tree hybrid zone. Gray represents high F_ST_ SNPs (portrayed as a histogram in **B**), included as a reference showing the cline distribution of parts of the genome likely under the strongest natural selection. Dashed red is moth frequency, black is Qscore, solid red is style length, solid blue is trunk height, and magenta is chloroplast haplotype frequency. **(A)** Full clines. All clines are oriented with the minimum on the left for clarity, which required flipping trait clines vertically. **(B)** Cline centers.

**FIGURE 5:**
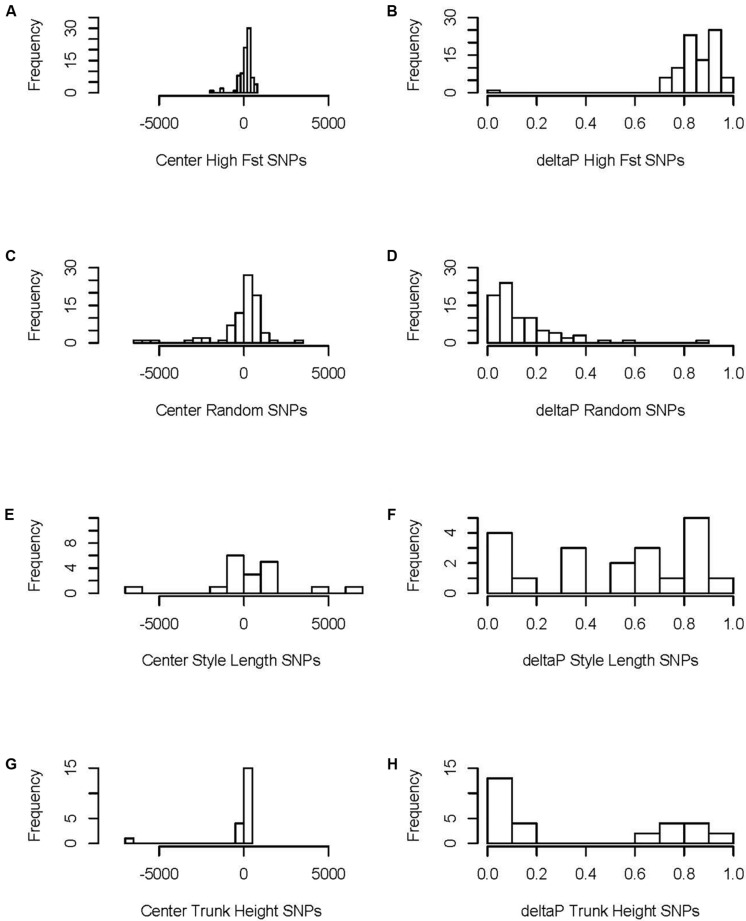
Distribution of cline parameters: cline centers (left) and delta P (the variation in allele frequency between the two ends of the hybrid zone, right) for high F_ST_ SNPs, randomly selected (non-high F_ST_) SNPs, and SNPs associated with style length and trunk height. The left column of the figure depicts the distribution of cline centers for SNPs, broken down by **(A)** high Fst, **(C)** random, **(E)** Style length, and **(G)** trunk height. The right column of the figure depicts the distribution of the difference between values on the extreme ends of the clines for SNPs, broken down by **(B)** high Fst, **(D)** random, **(F)** Style length, and **(H)** trunk height.

We found that the hybrid zone is delineated by a clear and narrow nuclear genomic center, whether it is measured using SNP-Qscore, high F_ST_ SNPs, or a random set of nuclear SNPs (Table 2 and Figures 4, 5). The frequency of different model types (Supplementary Table S2) reinforces this. As expected, the random SNPs have a much higher frequency of null clines than the other sets of SNPs, indicating that they are more likely to freely introgress (only one of the high-F_ST_ clines is a null, and only 5% of style length and 17% of trunk height SNPs). However, the majority of even random SNPs (80%) exhibit clines (all but one the simplest model) (Supplementary Table S2). The majority of high-F_ST_ clines (62%) and several trunk height and style length clines fit the more complex model II, with non-negligible proportion of high-F_ST_ clines fit best by model III (19%) (Supplementary Table S2). All of the asymmetric (model III) clines show longer introgression tails toward the west (see Appendix).

**TABLE 2:** Cline centers, maximums, and minimums (for the two ends of the cline) for non-SNP geographic clines (means of all SNP clines from the high-F_ST_ and random-SNP sets are included).

Trait	Best model	Center	Width	Center low estimate	Center high estimate	Pmin (west)	Pmax (right)
Style base width	II	–2265.88	10, 613.62	–4336.89	531.03	0.71	0.41
tree height	II	–2155.21	2702.79	–2284.25	–2065.65	0.81	0.50
Leaf width	II	–2038.1	11, 696.69	–3754.44	–1289.55	0.60	0.14
Cp haplotype	I	–852	276.40	–1036.3	–781.56	0.02	1.00
Mean random SNP	–	–124.22	961.96	–1945.28	1006.96	0.83	0.97
SNP Qscore	II	0	2236.39	–82.32	93.26	0.02	0.99
Trunk height	II	56.02	2139.12	49.81	118.1	0.10	0.40
Mean high F_ST_	–	105.85	2170.11	–193.77	404.56	0.12	0.98
Leaf length	III	157.37	1297.79	136.66	242.06	0.44	0.22
Style length	II	521.8	674.61	390.58	876.81	0.19	0.61
Moth	III	676.95	1229.31	585.21	676.95	0.00	0.99
Petal width	I	3251.09	6626.81	2280.26	5438.5	0.50	0.27

*Clines are listed in order of center location, from west to east. All measurements in meters. Low and high estimates for center are ± two log likelihoods of the mean, as produced by HZAR; for mean random SNP and mean high F_*ST*_ SNPs, the mean high and low estimates were used. For data on individual SNP clines, see Appendix. Traits for which linear regression across the cline was non-significant were excluded (branch number, petal length, pistil length, and pistil width). Pmin and Pmax for alleles are set with the minor allele frequency to the west; for phenotypes, pmin represents the west and pmax the eastern part of the distribution.*

The two morphological traits that vary most clearly between the species, trunk height and style length, have phenotypic clines that are closely coincident with the nuclear genomic clines, as does leaf length (Figure 4 and Tables 2, 3A). While the average of individual clines for SNPs associated in GWAS with trunk height and style length was coincident with the nuclear genomic clines, there was a lot of variation between loci; for style length in particular, some SNPs had clines with centers far from the hybrid zone center (Figure 5 and Supplementary Table S2). Comparing them to the mean high-F_ST_ cline shows the least consistency in the style SNPs, with half having centers significantly offset from the high-F_ST_ mean (one to the west, eight to the east). Trunk SNPs showed a more bimodal pattern, with one group fitting the high-F_ST_ pattern fairly well and the other diverging in similar ways to the style SNPs. Just over a third of the trunk height SNP clines had centers significantly offset from the high-F_ST_ mean (one to the west, five to the right). In addition to being more frequent, the aberrations in style SNPs are more extreme than those in trunk SNPs (Figure 5 and Appendix Table).

**TABLE 3:** Comparisons of geographic clines.

	Style base	Tree height	Leaf width	Chloroplast	Random SNP	SNP Qscore	Trunk height	High F_ST_	Leaf length	Style length	Moth	Petal width
Style base width	–	**Yes**	**Yes**	**Yes**	**Yes**	**Yes**	**Yes**	**Yes**	**Yes**	**Yes**	no	no
Tree height		–	**Yes**	No	**Yes**	No	No	No	No	No	no	no
Leaf width			–		No	No	No	No	No	No	no	no
Chloroplast				–	**Yes**	No	No	No	No	No	no	no
Mean random SNP					–	**Yes**	No	**Yes**	**Yes**	**Yes**	**yes**	no
SNP Qscore						–	**Yes**	**Yes**	No	No	no	no
Trunk height							–	**Yes**	No	No	no	no
Mean high F_ST_								–	**Yes**	No	no	no
Leaf length									–	No	no	no
Style length										–	**yes**	no
Moth											–	no
Petal width												–

*Cline coincidence, whether the cline centers are the same. Clines are listed in order of center location, from west to east. Cline comparisons with statistically indistinguishable parameters—that is to say, ones that are the same—are indicated with “yes” in bold.*

We found two chloroplast haplotypes in the hybrid zone, one clearly associated with each Joshua tree species, allowing us to perform a successful geographic cline analysis on this genotype. The cline center for chloroplast haplotype frequency is significantly offset from the nuclear genomic clines, and opposite the direction we predicted—to the west rather than the east (Table 2 and Figure 4). The chloroplast cline center is just east of the gap in the Joshua tree distribution, which is at most 2 km wide at the narrowest point with *Y. brevifolia* on the west and the hybrid zone to the east (Figure 3) (it may in fact be somewhat narrower, as we have not methodically sampled the edges). The cline is also startlingly steep, with only a single bin containing both genotypes (the bin directly adjacent to the gap, 1.07 km wide—with the exception of a single *Y. jaegeriana* haplotype appearing on the west side of the gap).

The phenotypic clines for other morphological traits deviated from the center of the hybrid zone in ways we did not predict. Tree height, style base width, and leaf width clines are offset in the same direction as the chloroplast haplotype cline, but even more dramatically (although the confidence intervals for estimating style base width cline center are so wide that they cross the center of the hybrid zone). A single morphological trait cline, petal width, is significantly offset to the east (Table 2).

Pollinator species abundances shift sharply in the center of the hybrid zone. The cline for moth species is, as predicted, most closely coincident with the style length cline. It also clusters with the nuclear genomic, phenotypic trunk height, and phenotypic leaf length clines (Figure 4 and Table 2).

The nuclear genomic clines together show a clear picture of higher genetic diversity (measured as more intermediate allele frequencies) in *Y. brevifolia* than in *Y. jaegeriana* (Figure 4).

### Cytonuclear Genotype Data

When we compare nuclear genotypes (summarized using Qscore) to chloroplast genotype, we see some dissociation of nuclear and cytoplasmic genomes, with *Y. jaegeriana* chloroplasts dominating in the hybrid zone. Because the sample size of trees genotyped at both SNPs and the chloroplast was small, we used Qscores calculated with microsatellite genotypes for the nuclear genome estimate (the same used to set the 0.5 hybrid index line for the clines) instead. This produced a set of 183 trees with data on cytonuclear genotypes (a distinct dataset, as not all of the trees with SNP genotypes had chloroplast genotypes and vice versa). Of the 43 trees in this set that are genomically hybrids (SNP Qscores between 0.15 and 0.85, Royer et al., 2016), only 21% (nine trees) have the *Y. brevifolia* chloroplast haplotype. There are no trees with Qscores above 0.44 that have a brevifolia chloroplast haplotype.

### Level and Pattern of Hybridization in the Zone

**FIGURE 6:**
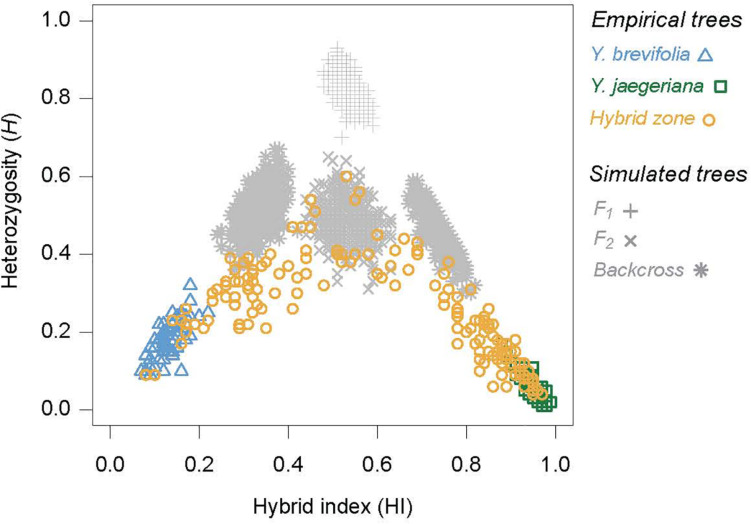
The joint distribution of hybrid index scores and heterozygosities for empirical and simulated trees based on the 101 highly differentiation loci. Empirical trees include pure individuals of both taxa, and individuals from inside the hybrid zone. The distribution of four simulated hybrid classes are shown in gray, including F_1_s, simulated by crossing the pure populations, F_2_s simulated by intercrossing the simulated F_1_s, and backcrosses, simulated by crossing the simulated F1s back to each parental type.

We estimated how many of the trees in our study were likely F1 hybrids, parental, backcrosses, or advanced generation hybrids (F2+) by plotting heterozygosity (*H*) on hybrid index score (HI), which revealed clear evidence for extensive genetic mixing between *Y. brevifolia* and *Y. jaegeriana* (Figure 6). Individuals from within the hybrid zone nearly spanned the full range of hybrid index scores, ranging from 0.14 to 0.97. Some individuals from the hybrid zone have genotypes consistent with being pure individuals of each species, as they overlap the clusters of *Y. brevifolia* and *Y. jaegeriana* from outside the hybrid zone. However, most individuals show some evidence of mixed ancestry. Even within the hybrid zone, the distribution of hybrid index scores was strongly clinal (Pearson correlation coefficient distance of hybrid index and geography = 0.66, *p* < 0.0001), so there may be little opportunity for pure individuals to mate with one another.

The distributions of simulated F_1_, F_2_ and backcross individuals provide deeper insight into the patterns and levels of hybridization between *Y. brevifolia* and *Y. jaegeriana.* For instance, none of the empirical hybrids had heterozygosities that were as high as is expected for an F_1_ hybrid, suggesting that they are absent from the zone. Very few individuals also overlapped the distributions of the first-generation backcrosses, mainly because their heterozygosities were lower than expected for these hybrid classes. Rather, most individuals are likely to be later generation backcrosses, or the results of mating between backcrosses and later-generation hybrids. However, some individuals overlap the distribution of simulated F_2_s, so may be early generation hybrids (Figure 6). Overall, these data indicate that individuals from this zone are highly mixed.

## Discussion

Our analysis of the Joshua tree hybrid zone found a narrow tension zone dominated by advanced generation hybrids (Figure 6), with largely coincident clines for tree genotype, phenotype, and pollinator occurrence (Figure 4). This is consistent with selection against hybrids resulting in moderate to strong reproductive isolation acting across the whole genome. The exception is the chloroplast cline, which was significantly offset to the west (opposite the direction predicted by previously documented gene flow) (Figure 4 and Table 2).

The concordance of the clines for moths and tree hybrid index is striking, with both being quite narrow, showing sudden and dramatic transitions in the center of the hybrid zone. The cline centers are offset, but overlap substantially over the geographic area of transition (width) in spite of steep transitions (Figure 4). There are two possible, non-mutually exclusive causes for the close fit of moth and tree clines. First, it may be that moth species perform poorly on non-native hosts, and do not disperse far. This is analogous to the classic definition of a tension zone (Barton and Hewitt, 1985), although in a pair of “good” species: moths may choose the “wrong” host readily, but such moths have fitness that is reduced (*T. antithethica*) or negligible (*T. synthetica*), reproducing little if at all. Moths would therefore rarely be found more than one generation’s dispersal distance from their host species. The other possibility is that they are excellent at detecting the correct host species, and their distribution mirrors that of the host because of behavior rather than selection-dispersal balance. Our statistical test suggests both mechanisms may be important, but the genetic identity of the tree is likely most important (this in spite of the strong correlation between genotype and location in the hybrid zone). Consistent with this, Smith et al. (2009) finds that pollinators show a strong preference for their host species, and that offspring survival on the wrong host is reduced at best. Preliminary data on *Tegeticula* movement in the hybrid zone show individual moths move an average of at least ∼30 meters (Autumn and Smith, unpublished data). Thus it seems likely that the matching clines of pollinator and host are due to a combination of choice, natural selection, and low dispersal.

Given the steep, narrow cline for pollinator species occurrence, we would expect strong selection from moths on style length to produce coincident and equally dramatic clines for the trait and correlated SNPs. Evidence for this is mixed. The phenotypic style length cline is coincident and concordant with the moth cline—in fact, the moth cline fits the style length cline better than it fits the hybrid index cline, and style length is the only phenotypic cline for which both the center and width are statistically indistinguishable from that of moths (Table 3). However, the clines for SNPs associated with variation in style length do not consistently show the same pattern, and include loci with cline centers significantly displaced from the moth cline in both directions (Figure 5 and Supplementary Table S2). The relatively low number of SNPs identified through RADseq means it is likely that we missed selection on many parts of the genome, making our pool of SNPs correlated with style length smaller than it should be (Lowry et al., 2017a, b). However, there is no reason to suspect this would bias our sample in favor of weaker correlations or false positives. On the other hand, if our threshold for significance in the GWAS was too low, it would lead to the presence of false positives in the style SNP pool. This could explain why the SNP clines are so much more diffuse than the phenotypic cline for style length. Regardless, given the data we have, our genotypic clines are not consistent with strong selection on style length in the hybrid zone.

When all the clines are viewed as a whole, the main theme is overall coincidence of narrow genomic clines (including random SNPs not under selection as well as Qscore and high-F_ST_ SNPs) (Figure 5). This is characteristic of tension zones, where hybrids are less fit than parents and selection is acting against hybrids on many different parts of the genome (Key, 1968; Barton, 1979; Barton and Hewitt, 1985) (Table 2). The comparison of hybrid index to heterozygosity reinforces this; first-generation hybrids are completely absent in our sample, and F2s are rare. Most of the hybrids are backcrosses and advanced-generation hybrids (Figure 6). These trends are all consistent with previous high F_ST_ estimates, confirming the species are highly differentiated (Royer et al., 2016). The inability to identify particular targets or agents of selection is a predictable side effect of this phenomenon.

We found higher genetic diversity in *Y. brevifolia*, based on higher rates of allele fixation in *Y. jaegeriana* across all nuclear markers (i.e., higher minor allele frequencies in *Y. brevifolia*) (Figures 4A, 5). This could be caused by introgression into *Y. brevifolia*, differences in effective population size, or differential maintenance of ancestral polymorphism. Previous population genetic work using microsatellites has suggested gene flow into *Y. brevifolia* (Starr et al., 2013), and we see evidence of introgression in the same direction in the clines—the few asymmetrical (model 3) clines (19% of the high-F_ST_ SNP pool) show introgression into *Y. brevifolia* (Supplementary Table S3), in the same direction that moth movement and population genetic data suggest gene flow should move (Smith et al., 2009; Starr et al., 2013).

The exception to the overall coincidence of clines, and perhaps our most surprising result, is the chloroplast cline center that is offset from nuclear clines opposite the direction we predicted (Figure 4 and Table 2). Cytonuclear discordance is generally common in hybrid zones, but diagnosing the cause is challenging (Toews and Brelsford, 2012). The three basic mechanisms of all short-term evolution are the same ones that can explain a mismatch between cytoplasmic and nuclear genomes in hybrid zones: natural selection, drift, or gene flow (Arnold, 1993).

If natural selection is causing the introgression of *Y. jaegeriana* chloroplasts into *Y. brevifolia*, introgression could be due to the *Y. jaegeriana* haplotype being inherently superior, the *Y. jaegeriana* haplotype being better adapted locally (e.g., Sambatti et al., 2008; Campbell et al., 2010), or cytonuclear interactions in hybrids with cytoplasm from one parent and nuclear genome from the other favoring the spread of the *Y. jaegeriana* haplotype. We have no data to test global superiority of one cytoplasmic genome in this system. Previous niche modeling has found little or no difference in the ecological niches of *Y. brevifolia* and *Y. jaegeriana* in and around the hybrid zone (Godsoe et al., 2009), so we have little support for local adaptation favoring one haplotype on the east side of the hybrid zone and another on the west. The last possible source of selection, cytonuclear interactions, commonly affect fitness (Burke and Arnold, 2001; Toews and Brelsford, 2012; Dobler et al., 2014), particularly in hybrids (Dobler et al., 2014), where they frequently appear in the form of cytoplasmic male sterility (CMS) (Frank, 1989). If interactions between the *Y. jaegeriana* cytoplasmic genome and the *Y. brevifolia* nuclear genome result in reduced male fitness, it could cause increased female fitness in hybrid plants with the *Y. jaegeriana* cytoplasmic genome, leading to the spread of that haplotype (Tsitrone et al., 2003). Such patterns of chloroplast capture have been shown to be widespread in plants (Rieseberg and Soltis, 1991; Bock et al., 2014) and would be sufficient to create the pattern we see in the Joshua tree hybrid zone.

Either drift or gene flow can also contribute to cytonuclear discordance, but neither seems likely to be responsible for the offset centers in the Joshua tree hybrid zone. Drift can shape clines of loci not under strong selection, as evidenced by the wider range of centers in our randomly selected set of low-F_ST_ SNP clines (centers range from −6573 to 2945, with 11% having centers farther from the center of the hybrid zone than the chloroplast cline; Table 2 and Supplementary Table S2). Cytoplasmic genomes, with effective population sizes 1/4 that of the nuclear genome (Birky et al., 1983), are particularly prone to drift (Hudson and Turelli, 2003). However, because we have evidence from ecological and population genetic data that the chloroplast introgression in the Joshua tree hybrid zone is happening in spite of nuclear gene flow that should oppose it (compare our result with Smith et al., 2009 and Starr et al., 2013), we conclude selection is most likely responsible for the western displacement of the chloroplast cline center.

The cytonuclear genotypes present in the hybrid zone suggest cytonuclear incompatibilities may well play a role. With the large number of advanced hybrids and backcrosses present (Figure 6), the hybrid swarm should create opportunities for nuclear and cytoplasmic parental genomes to become dissociated, allowing chloroplast introgression to potentially happen in either direction. In the absence of selection, observed pollinator preferences would still push chloroplast introgression into *Y. jaegeriana* rather than what we observe. Instead, we find only partial disassociation of chloroplast and nuclear genomes. Opposite our prediction, most hybrids (∼80%) carry the *Y. jaegeriana* chloroplast haplotype. In addition, while all trees with genomic compositions consistent with backcrossing to *Y. jaegeriana* (Qscore > 0.5) have *Y. jaegeriana* chloroplast haplotypes, those with Qscores consistent with backcrossing to *Y. brevifolia* are narrowly dominated by the *Y. jaegeriana* chloroplast haplotype as well (58%). While far from decisive, these data suggest a role for selection and reinforce that gene flow cannot explain the chloroplast cline.

The explicitly geographic approach of our analysis highlights a significant feature of the hybrid zone relevant to understanding gene flow that has been ignored in previous studies: the ancient, dry lakebed that causes a roughly 1km gap in the distribution of Joshua trees just west of, and roughly parallel to, the center of the hybrid zone (Figure 3). The very low density of trees in this zone (there are a few, unsampled, individuals scattered throughout it) could be caused by sandier or saltier soil resulting in moisture levels too low for Joshua trees to tolerate. Across their range, Joshua trees are typically absent from pluvial lakebeds (C. Smith, personal observation), which are often dominated by *Atriplex canescens*, a highly salt tolerant species of salt bush that is widespread throughout western North America (Glenn et al., 1996). In fact, the entire hybrid zone is located east of the lakebed, with the nearest individuals west of the gap considered part of a population categorized as *Y. brevifolia* rather than hybrid (Starr et al., 2013), and all RADseq-genotyped individuals having Qscores below 0.06. This suggests that gene flow across the gap is relatively rare. That would be consistent with what we know about gene flow in Joshua trees; estimates of seed dispersal are around 30.0 ± 16.8m from the maternal tree (Vander Wall et al., 2006), and measurements of movement in other species of *Tegeticula* estimate moths move less than 50 m (Marr et al., 2000); as mentioned above, preliminary work in Joshua tree *Tegeticula* shows individual moths move an average of ∼30 m (31.79 ± 34.17 m; Autumn and Smith, unpublished data) (although this is likely an underestimate, Smith pers. comm.). This is roughly similar to estimates of *Yucca filamentosa* pollen dispersal by its moth pollinator, *Tegeticula yuccasella*: mean 4.66 ± 10.23 m (Marr et al., 2000). Occasional long-distance dispersal events certainly occur, but at a scale of >30x the mean, they may be quite rare.

It is likely that this area of low population density is the feature anchoring the hybrid zone in its current location. Movement of hybrid zones is generally a common feature; although many tension zones are currently stable, others have been shown to move (Buggs, 2007; Wielstra et al., 2017; van Riemsdijk et al., 2019). Directional hybrid zone movement can be in response to asymmetric gene flow, because one species is favored by natural selection, or in response to differences in population density (Buggs, 2007). Movement toward areas of low population density has long been thought to be a predominant driver of location and a consistent feature of tension zones (Barton, 1979; Barton and Hewitt, 1985). After being pulled toward an area of low population density, a hybrid zone will stop altogether and be held stationary if it reaches an uninhabitable or extreme low density region (Barton and Hewitt, 1985). The distribution gap formed by the dry lakebed west of the Joshua tree hybrid zone meets these criteria.

If the Joshua tree hybrid zone originally formed substantially east of its current location and subsequently moved in response to low density to the west, we would expect to see asymmetric clines with longer tails to the east across the genome, signaling greater introgression where the center of the hybrid zone used to be (Barton and Hewitt, 1985; Currat et al., 2008; Wielstra et al., 2017; van Riemsdijk et al., 2019). For clines with neutral variation, the centers should also be offset to the east, including the cytoplasmic genome (assuming selection is not acting on it, which is obviously a big assumption—see above) (Wielstra et al., 2017). This signal of introgression is expected to linger indefinitely (Currat et al., 2008). In fact, we see none of these features in the Joshua tree hybrid zone; all nuclear clines, including those for loci not under selection, share roughly the same center. The most likely history for the Joshua tree hybrid zone is what Barton (1979) believed to be the predominant model—the hybrid zone barely moved from the point where it first arose before being anchored long-term as a tension zone next to an area of low population density, the ancient lakebed on the west side of Tikaboo Valley.

## Conclusion

We find that the patterns we expected to see based on previous ecological and population genetic work in and around the hybrid zone were not fully supported: the Joshua tree hybrid zone appears to be dominated by advanced-generation hybrids, is most strongly shaped by selection against early-generation hybrids, and displays a chloroplast cline displaced to the west (opposite our prediction). Evidence for direct phenotypic selection from pollinators is weak by comparison. These results suggest several new clear avenues for further work. First, a focus on how pollinators interact with advanced-generation hybrids (pollinator choice and performance). Previous work focused on reproductive isolation, comparing performance of pollinators on the opposite host. In fact, it seems that the formation of F1 hybrids is quite rare, so the movement of pollen between advanced generation hybrids (particularly backcrossed ones) and parentals is much more relevant to understanding gene flow in the current hybrid zone. In addition, the clear and unexpected pattern of chloroplast haplotype displacement to the west sets up several testable alternate hypotheses. The work described above on pollinator behavior may reveal pollen movement patterns that differ from those described in crosses between pure parental plants, consistent with chloroplast introgression. Additional work quantifying investment in male and female reproduction by hybrids with different chloroplast haplotypes (ovule and pollen counts) should reveal whether cytonuclear incompatibility is lowering overall fitness in hybrids with the *brevifolia* chloroplast haplotype, or increasing female fitness of those with the *jaegeriana* chloroplast haplotype. Finally, fully understanding the barriers contributing to reproductive isolation in Joshua trees will require going beyond population genomic methods to experimental approaches quantifying each barrier, prezygotic and postzygotic, to reproduction across these species (Sobel and Chen, 2014).

## Data Availability Statement

The original contributions presented in the study are included in the article/Supplementary Material. Further inquiries can be directed to the corresponding author.

## Author Contributions

AR contributed to design, performed the analyses, and wrote the manuscript. JW-H collected and genotyped moths. CS provided phenotypic and genomic data, funding, and input into writing and interpretation.

## Conflict of Interest

The authors declare that the research was conducted in the absence of any commercial or financial relationships that could be construed as a potential conflict of interest.

## Footnotes

1 www.codoncode.com
