# Supplementary figures and images for: Strong Selection Against Early Generation Hybrids in Joshua Tree Hybrid Zone Not Explained by Pollinators Alone
Source: Front Plant Sci. 2020 May 26;11:640. doi: 10.3389/fpls.2020.00640 (PMC7264850; doi:10.3389/fpls.2020.00640)

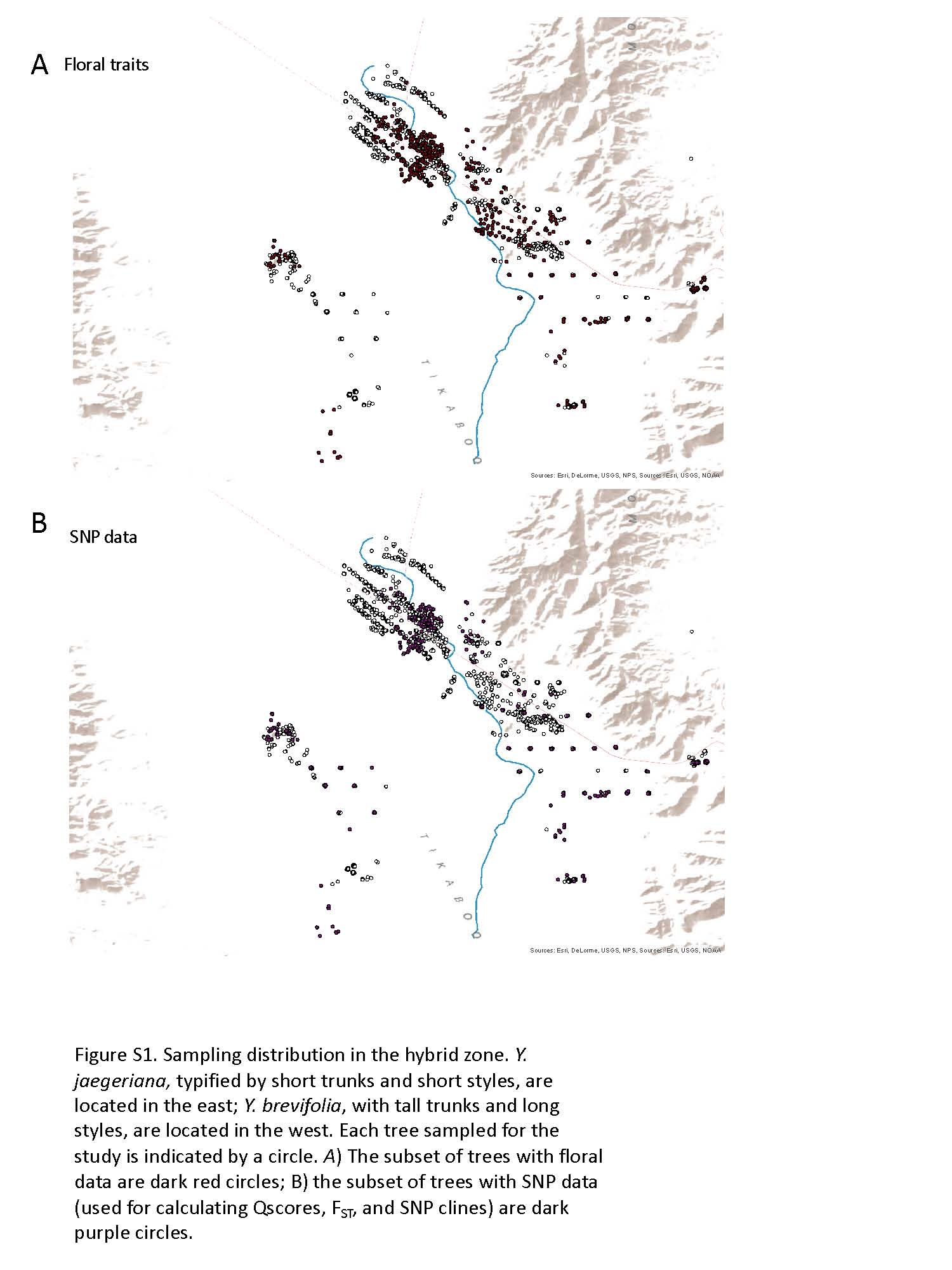

Supplement: Supplementary file 2 [file Image_1.JPEG]
