# Supplementary material for: Strong Selection Against Early Generation Hybrids in Joshua Tree Hybrid Zone Not Explained by Pollinators Alone
Source: Front Plant Sci. 2020 May 26;11:640. doi: 10.3389/fpls.2020.00640 (PMC7264850; doi:10.3389/fpls.2020.00640)
Supplement: Supplementary file 3 [file Table_1.docx]

Table S1. Trees sampled per bin in the geographic clines analysis (from the total of 19 bins). The SNP category covers SNP Qscores as well as high F_ST_, random SNPs, and analyses of loci associated with trait variation. For moths, multiple moths were sampled per tree (see methods).

| **Trait** | **# trees** | **mean per bin** | **min per bin** | **max per bin** |
| --- | --- | --- | --- | --- |
| *VEGETATIVE*  branch number | 2881 | 151.63 | 37 | 487 |
| height | 2921 | 153.74 | 37 | 487 |
| leaf length | 2917 | 153.53 | 37 | 486 |
| leaf width | 2929 | 154.16 | 37 | 486 |
| trunk height | 2937 | 154.58 | 37 | 487 |
| *FLORAL*  petal length | 521 | 27.42 | 4 | 51 |
| petal width | 521 | 27.42 | 4 | 51 |
| pistil length | 525 | 27.63 | 4 | 51 |
| pistil width | 525 | 27.63 | 4 | 51 |
| style base width | 525 | 27.63 | 4 | 51 |
| style length | 525 | 27.63 | 4 | 51 |
|  |  |  |  |  |
| *GENETIC*  chloroplast | 205 | 10.79 | 4 | 28 |
| SNP Qscore | 308 | 16.21 | 5 | 38 |
| *POLLINATORS*  Moth sp. frequency | 101 | 5.32 | 3 | 12 |

Table S2. Model parameters for SNP frequency clines in Tikaboo Valley. Model type was assessed using AIC; if a more complex model had a lower AIC but was within two points of the less complex model, the cline was assigned to the simpler model. Random SNPs were randomly selected from all available SNPs; the Trunk height and Style length SNPs were those that were significantly associated with phenotypic variation in those traits in a GWAS. Note that there is some overlap in SNP categories; e.g., one SNP present in the Trunk height category is also in Style length.

|  | |  |  |  | |  | | |  |  |  |  |  |  |  |  |
| --- | --- | --- | --- | --- | --- | --- | --- | --- | --- | --- | --- | --- | --- | --- | --- | --- |
|  |  | Model type frequency | | | | | |  | | |  | |  | |  | |
| SNP type | *n* | null | I | II | III | | mean center | | | sd center | | mean width | | sd width | |  |
| random | 100 | 20 | 79 | 1 | 0 | | -113.89 | | | 1464.27 | | 958.69 | | 1463.99 | |  |
| high Fst | 84 | 0.01 | 0.18 | 0.62 | 0.19 | | 105.85 | | | 398.33 | | 2170.11 | | 1519.45 | |  |
| Trunk height | 30 | 0.17 | 0.76 | 0.07 | 0 | | 24.74 | | | 1566.41 | | 1399.92 | | 1863.24 | |  |
| Style length | 19 | 0.05 | 0.63 | 0.32 | 0 | | 516.14 | | | 2558.81 | | 5976.19 | | 4390.98 | |  |

Table S3. Comparisons of geographic cline width. Clines are listed in order of center location, from west to east. A) Estimate of width, with low and high estimates a distance of two log-likelihoods from the best estimate. B) Concordance, whether the widths are the same (>/< signs refer to the row, then column; e.g the tree height cline is narrower than the leaf width cline, so the cell with height as row and leaf width as column is ‘<’.

A.

| trait | width | Width low estimate | Width high estimate |  |
| --- | --- | --- | --- | --- |
| Style Base Width | 10613.62 | 4660.16 | 13099.44 |  |
| Tree Height | 2702.79 | 2702.79 | 2742.59 |  |
| Leaf Width | 11696.69 | 7458.55 | 12887.30 |  |
| Cp Haplotype | 276.40 | 10.82 | 1219.80 |  |
| Mean random SNP | 961.96 | 192.96 | 5999.21 |  |
| SNP Qscore | 2236.39 | 2059.93 | 2427.013 |  |
| Trunk Height | 2139.12 | 1869.70 | 2187.72 |  |
| Mean high F_ST_ | 2170.11 | 1256.42 | 3469.76 |  |
| Leaf Length | 1297.79 | 1078.28 | 1457.83 |  |
| Style Length | 674.61 | 379.16 | 3965.41 |  |
| Moth | 1229.31 | 1229.31 | 1365.89 |  |
| Petal Width | 6626.81 | 4798.146 | 9918.76 |  |

| B. |  |  |  |  |  |  |  |  |  |  |  |  |
| --- | --- | --- | --- | --- | --- | --- | --- | --- | --- | --- | --- | --- |
|  | style base | tree height | leaf width | chloroplast | random SNP | SNP Qscore | trunk height | high F_ST_ | leaf length | style length | moth | petal width |
| style base width | - | > | = | > | = | > | > | > | > | > | > | = |
| tree height |  | - | < | > | = | > | > | = | > | = | > | < |
| leaf width |  |  | - | > | > | > | > | > | > | > | > | = |
| chloroplast |  |  |  | - | = | < | < | < | = | = | < | < |
| mean random SNP |  |  |  |  | - | = | = | = | = | = | = | = |
| SNP Qscore |  |  |  |  |  | - | = | = | > | = | > | < |
| trunk height |  |  |  |  |  |  | - | = | > | = | > | < |
| mean high F_ST_ |  |  |  |  |  |  |  | - | = | = | = | < |
| leaf length |  |  |  |  |  |  |  |  | - | = | = | < |
| style length |  |  |  |  |  |  |  |  |  | - | = | < |
| moth |  |  |  |  |  |  |  |  |  |  | - | < |
| petal width |  |  |  |  |  |  |  |  |  |  |  | - |
